# The exocyst complex in neurological disorders

**DOI:** 10.1007/s00439-023-02558-w

**Published:** 2023-04-22

**Authors:** Dilara O. Halim, Mary Munson, Fen-Biao Gao

**Affiliations:** 1 Department of Neurology, University of Massachusetts Chan Medical School, Worcester, MA USA; 2 Graduate Program in Neuroscience, Morningside Graduate School of Biomedical Sciences, University of Massachusetts Chan Medical School, Worcester, MA USA; 3 Department of Biochemistry and Molecular Biotechnology, University of Massachusetts Chan Medical School, Worcester, MA USA

## Abstract

Exocytosis is the process by which secretory vesicles fuse with the plasma membrane to deliver materials to the cell surface or to release cargoes to the extracellular space. The exocyst—an evolutionarily conserved octameric protein complex—mediates spatiotemporal control of SNARE complex assembly for vesicle fusion and tethering the secretory vesicles to the plasma membrane. The exocyst participates in diverse cellular functions, including protein trafficking to the plasma membrane, membrane extension, cell polarity, neurite outgrowth, ciliogenesis, cytokinesis, cell migration, autophagy, host defense, and tumorigenesis. Exocyst subunits are essential for cell viability; and mutations or variants in several exocyst subunits have been implicated in human diseases, mostly neurodevelopmental disorders and ciliopathies. These conditions often share common features such as developmental delay, intellectual disability, and brain abnormalities. In this review, we summarize the mutations and variants in exocyst subunits that have been linked to disease and discuss the implications of exocyst dysfunction in other disorders.

## Introduction

**Fig. 1:**
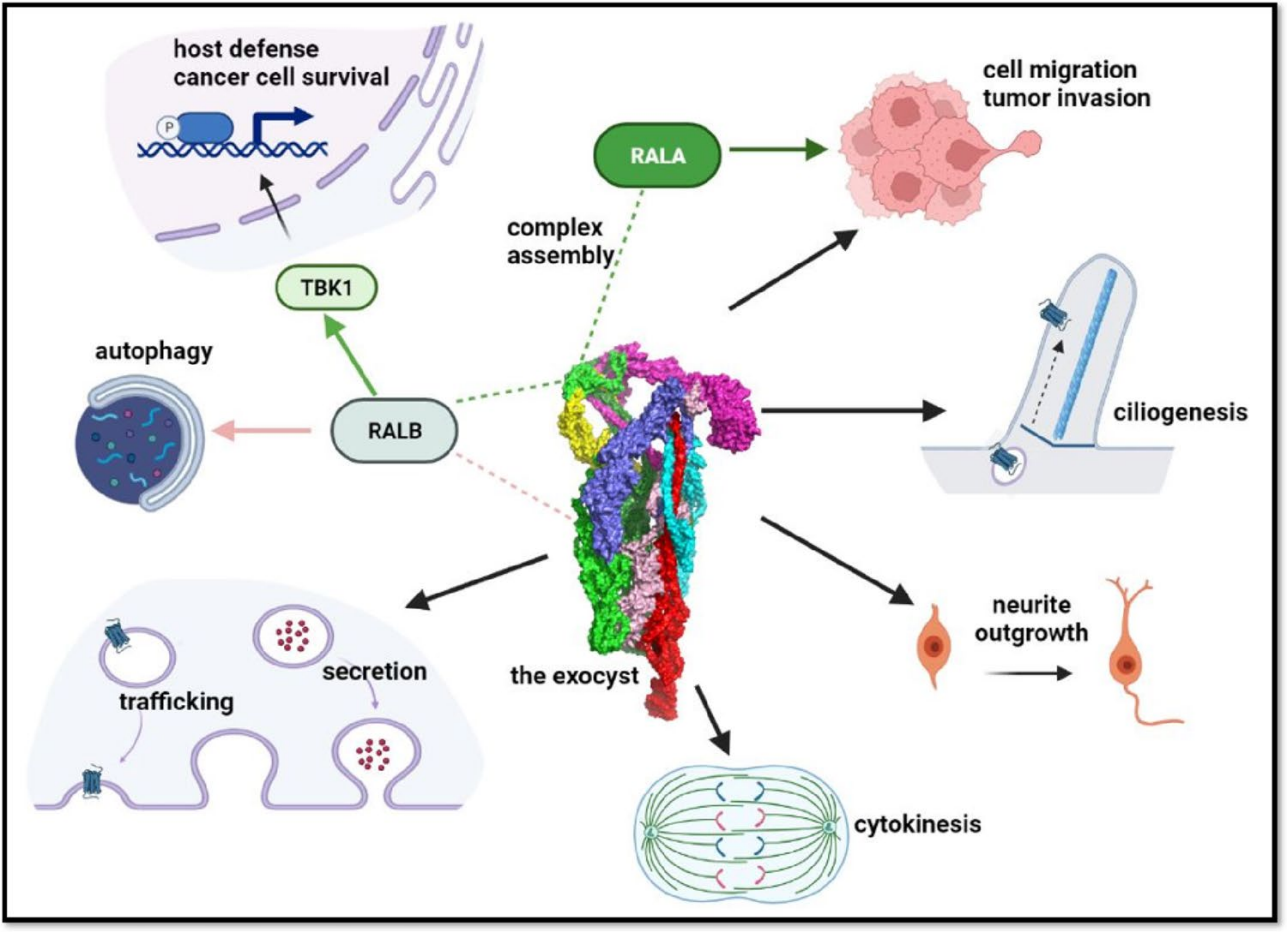
Diverse functions of the exocyst. The exocyst, an octameric complex consisting of EXOC1-8 subunits, functions as an effector of RALA and RALB GTPase signaling mediated by EXOC2 and EXOC8 binding. The interaction with RALA is also important for the assembly of the complex. The exocyst has diverse roles, including protein trafficking, vesicle tethering to the plasma membrane for secretion, cytokinesis, neurite outgrowth, and ciliogenesis. Through its interaction with RALA, the exocyst also participates in cell migration and tumor invasion. The interaction between EXOC8 and RALB regulates autophagy initiation, and EXOC2 and RALB together serve as a scaffold to recruit and activate TBK1 for host defense and survival of cancer cells. EXOC1: yellow, EXOC2: light green, EXOC3: magenta, EXOC4: dark green, EXOC5: cyan, EXOC6: red, EXOC7: dark blue, EXOC8: pink. Partially created with BioRender.com

Exocytosis refers to the process of the fusion of secretory vesicles with the plasma membrane to deliver materials to the cell surface or to release cargoes to the extracellular space. Vesicle fusion with the plasma membrane is driven by SNARE proteins on the vesicles and the plasma membrane. The exocyst mediates the spatial and temporal regulation of SNARE complex assembly for fusion and the tethering of the secretory vesicles to the plasma membrane before fusion (Heider and Munson 2012). The exocyst—an octameric protein complex comprising Sec3 (EXOC1), Sec5 (EXOC2), Sec6 (EXOC3), Sec8 (EXOC4), Sec10 (EXOC5), Sec15 (EXOC6), Exo70 (EXOC7), and Exo84 (EXOC8) subunits—engages in diverse cellular functions, including incorporation of proteins and lipids to plasma membrane, cell polarity, neurite outgrowth, ciliogenesis, cytokinesis, cell migration, autophagy, host defense, and tumorigenesis (Fig. 1). The role of the exocyst is discussed in detail elsewhere (Heider and Munson 2012; Wu and Guo 2015; Martin-Urdiroz et al. 2016; Tanaka et al. 2017).

The exocyst fulfills important functions in neurons, which are highly polarized cells with complex dendritic and axonal morphology and synaptic structure. The exocyst regulates dendrite and axon outgrowth through cytoskeletal reorganization and membrane expansion. Neurite outgrowth defects have been observed in cultured primary neurons, neuronal cell lines, and flies with dysfunctional exocyst subunits (Hazuka et al. 1999; Vega and Hsu 2001; Murthy et al. 2003; Lira et al. 2019; Plonka et al. 2021; Swope et al. 2022). Neurons also use a specialized form of exocytosis called synaptic transmission. However, mutations in the exocyst do not affect synaptic vesicle fusion in flies (Murthy et al. 2003). Thus, the exocyst appears to have varying degrees of involvement in diverse types of exocytic pathways.

First identified in yeast, the exocyst is evolutionarily conserved from plants to flies and mammals (Novick and Schekman 1979; Ting et al. 1995; TerBush et al. 1996; Elias et al. 2003; Murthy et al. 2003). Exocyst subunits have also gained new and diverse functions in mammals (for a review, see Tanaka et al. 2017). Exocyst subunits are essential for viability, and complete loss of each of multiple subunits is lethal in yeast, flies, and mice (Novick and Schekman 1979; Friedrich et al. 1997; Murthy et al. 2003, 2005; Fogelgren et al. 2015; Mizuno et al. 2015).

Recent advances in cryo-electron microscopy have helped resolve the structure of exocyst subunits and provided better insights into how the complex assembles in yeast. Structural studies and cell biological data suggest stepwise pairing interactions through CorEx domains, the core exocyst assembly domains, between subunits Sec3–Sec5, Sec6–Sec8, Sec10–Sec15, and Exo70–Exo84 to form heterodimers. Two CorEx pairs then assemble into subcomplex I (Sec3–Sec5–Sec6–Sec8) and subcomplex II (Sec10–Sec15–Exo70–Exo84), which interact to form the holocomplex. The structure and assembly of the exocyst complexes and vesicle tethering are discussed in detail elsewhere (Mei et al. 2018; Mei and Guo 2019; Lepore et al. 2018).

In metazoans, the exocyst is also an effector of the RAL GTPases RALA and RALB; this function is mediated through the binding of Sec5 and Exo84 (Moskalenko et al. 2002, 2003; Chien et al. 2006). RAL GTPases belong to RAS superfamily of small GTPases and share high structural similarity with RAS GTPases. Both RALA and RALB are the effectors of RAS signaling, and promote tumor proliferation, survival, and metastasis in diverse human cancers with RAS mutations. The interaction with RALA is important for exocyst assembly and function in exocytosis, and the interaction with RALB is important for cellular signaling via TBK1 in host defense and the oncogenic response (Fig. 1) (reviewed in Bodemann and White 2008; Yan and Thedorescu 2018).

Mutations or variants in exocyst subunits occur in patients with other diseases, especially neurodevelopmental disorders, causing developmental delay, intellectual disability, and brain abnormalities. Several exocyst subunits have also been implicated in ciliopathies, a class of genetic disorders linked to mutations in genes affecting cilia function. Ciliopathies have a core feature of neurodevelopmental defects, along with some other multi-organ manifestations such as kidney and liver diseases, skeletal malformation, and retinitis pigmentosa (reviewed in Badano et al. 2006; Reiter and Leroux 2017).

Multiple exocyst subunits have been implicated in the pathogenesis of various human diseases such as polycystic kidney disease, cancer, and AIDS (reviewed in Martin-Urdiroz et al. 2016). Here, we summarize the mutations and variants in exocyst subunits that have been particularly linked to neurodevelopmental disorders and ciliopathies (Table 1).

**Table 1:** Human diseases linked to mutations and variants in the exocyst subunits and RALA

Gene	Disease (OMIM #)	Reference
*EXOC2*	Neurodevelopmental disorder with dysmorphic facies and cerebellar hypoplasia (OMIM **#**619306)	Van Bergen et al. (2020)
*EXOC3L2*	Meckel–Gruber-like syndrome	Shaheen et al. (2016)
	Novel syndrome with Dandy–Walker malformation	Shalata et al. (2019)
*EXOC4*	Meckel–Gruber syndrome	Shaheen et al. (2013)
*EXOC6B*	Spondylo-epi-metaphyseal dysplasias with joint laxity (SEMDJL, type 3 OMIM #618395)	Girisha et al. (2016)
		Campos-Xavier et al. (2018)
		Simsek-Kiper et al. (2022)
	Complex syndromes including intellectual disability*	Borsani et al. (2008)
		Fruhmesser et al. (2013)
		Wen et al. (2013)
		Evers et al. (2014)
*EXOC7*	Neurodevelopmental disorder with seizures and brain atrophy (OMIM #619072)	Coulter et al. (2020)
*EXOC8*	Neurodevelopmental disorder with microcephaly, seizures, and brain atrophy (OMIM #619076)	Coulter et al. (2020)
		Ullah et al. (2022)
	Joubert syndrome*	Dixon-Salazar et al. (2012)
*RALA*	Hiatt–Neu–Cooper neurodevelopmental syndrome (OMIM #619311)	Hiatt et al. (2018)
		Tanaka et al. (2017)
		Okamoto et al. (2019)

*The involvement of the variant in the disorder has not been confirmed

### *EXOC2*

The first three patients carrying pathogenic variants in *EXOC2 (Sec5)* were identified in two independent families (Van Bergen et al. 2020) (Table 1). These patients had severe developmental delay, facial dysmorphism, and brain abnormalities. Two affected children in a consanguineous family were homozygous for the c.1309C > T; p.Arg437* nonsense variant and had microcephaly and spastic quadriplegia. One affected child in a nonconsanguineous family was compound heterozygous for the c.389G > A; p.Arg130His and c.1739T > C; p.Leu580Ser missense variants and had milder clinical symptoms. The c.1309C > T variant leads to a frameshift and a premature stop codon, resulting in nonsense-mediated decay of *EXOC2* mRNA and production of a truncated EXOC2 protein. Fibroblasts from the patients with the c.1309C > T variant had no full-length protein. Although complete knockout of *EXOC2* is expected to be embryonic lethal, the low level of truncated EXOC2 in these patients was sufficient to support cell viability. The c.1309C > T variant retains the CorEx domain, which is important for the interaction with the other exocyst subunits, suggesting that the truncated EXOC2 protein was at least partially functional. This finding is supported by the viability of *Drosophila* with truncated Sec5/EXOC2 retaining the CorEx domain (Sommer et al. 2005). Further characterization of the patient cells also revealed reductions in exocytosis and the vesicle fusion rate and defects in Arl13b localization to primary cilia. On the other hand, in cells from the patient with the compound heterozygous variant, exocytosis was reduced without any significant changes in the EXOC2 level or vesicle fusion rate, which can explain the milder clinical symptoms. The findings in this study highlight the importance of exocyst function and exocytosis in brain development by linking exocyst subunit EXOC2 to neurodevelopmental disorders. Further studies to elucidate the structure and function of EXOC2 will clarify the complex role of the exocyst in neuron biology.

### *EXOC3L2*

Analysis of a large cohort of patients with ciliopathy spectrum revealed novel candidate genes for ciliopathies, including *EXOC3L2* (Shaheen et al. 2016) (Table 1). A patient who was homozygous for the c.398dupC; p.Leu134Thrfs*25 variant in *EXOC3L2* had symptoms resembling those of Meckel-Gruber syndrome (MKS), including occipital encephalocele and early postnatal death, but lacked polydactyly and enlarged cystic kidneys, two other criteria for MKS. The mutation was predicted to cause loss of function of EXOC3L2 and considered to be the pathological variant.

In another study, novel mutations of *EXOC3L2* were found in two families with Dandy–Walker malformation (DWM) (Shalata et al. 2019) (Table 1), which primarily affects cerebellar development and is often associated with ciliopathies. One family was consanguineous and had three aborted fetuses, all diagnosed with DWM, carrying the homozygous c.122T > A; p.Leu41Gln mutation in *EXOC3L2*. Another patient from an independent consanguineous family was homozygous for the p.Arg72* nonsense mutation. The affected child had DWM with additional symptoms including renal dysplasia and bone marrow failure. Fibroblasts from the patient showed that the p.Arg72* truncation mutation resulted in marked reduction in EXOC3L2 level. This study was the first to link DWM and the exocyst and highlighted an important role for the exocyst in the nervous system as well as in kidneys and bone marrow. Kidney failure in patients with a dysfunctional exocyst complex can be explained by its role in ciliogenesis. However further studies are needed to explain the role of the exocyst in bone marrow failure.

### *EXOC4*

Characterization of a small MKS cohort to identify novel candidate MKS genes revealed the first potentially pathogenic variant in *EXOC4 (Sec8/Sec8L1*) that causes MKS (Shaheen et al. 2013) (Table 1). MKS, a severe form of ciliopathy, is characterized mainly by early lethality, occipital encephalocele, enlarged cystic kidneys, and polydactyly and is often associated with additional symptoms, including brain abnormalities. The affected individual was homozygous for the c.1733A > G; p.Gln578Arg variant in *EXOC4* and had all of the main features of MKS along with microcephaly and several head and neck malformations. MKS is a genetically heterogeneous disease and the MKS genes that have been identified so far play a role in cilia biology. Given the role of the exocyst in ciliogenesis, the novel variant of *EXOC4* is likely to be a causative variant in MKS. MKS is one of the more severe ciliopathies, and most causative mutations are protein truncating (Salonen et al. 2011). Although the p.Gln578Arg is a missense variant, it is not known how it may lead to severe form of MKS. Studies of the effect of the variant on the structure and level of EXOC4 and its interaction with other exocyst subunits might explain the severity of the phenotype with the missense variant.

### *EXOC6B*

Pathogenic variants in *EXOC6B (Sec15B)* have been identified in six patients with spondylo-epi-metaphyseal dysplasias with joint laxity (SEMDJL) (Girisha et al. 2016; Campos-Xavier et al. 2018; Simsek-Kiper et al. 2022) (Table 1), a skeletal disorder characterized by spine deformities, severe joint laxity, and multiple joint dislocations. The first two patients were from a consanguineous family with variants in *EXOC6B* with SEMDJL (Girisha et al. 2016). The patients were homozygous for the c.906T4A; p.Tyr302* nonsense variant, which was considered the most likely cause. In a subsequent study, two patients from a family with SEMDJL (Campos-Xavier et al. 2018) had the c.915 + 20070_2197-135947del; p. Gly305_Gln732 del variant, which was considered to be pathogenic. One of them also had intellectual disability, but it remains unknown whether the *EXOC6B* variant was responsible. Recently, another study reported two patients with SEMDJL from unrelated consanguineous families (Simsek-Kiper et al. 2022). One was homozygous for the c.2122 + 15447_2197-59588del; p.Gln708Profs*16 variant and the other was homozygous for the c.401T > G; p.Leu134* variant in *EXOC6B*. The patient carrying the p.Leu134* variant also had developmental delay, intellectual disability, and brain abnormalities, which had never been reported in SEMDJL. Considering the role of the exocyst in neuron biology, these neurological defects might be related to the early truncation of EXOC6B and the severity of the mutation. In fibroblasts derived from both patients, the EXOC6B level was significantly reduced, exocytosis was impaired, and primary cilia length was shortened. This study was the first to link exocytosis, ciliogenesis, and SEMDJL pathology (Simsek-Kiper et al. 2022).

EXOC6B has also been implicated in the pathogenesis of intellectual disability in four studies (Table 1). The first patient had a complex phenotype, including renal deficiency, neutropenia, recurrent pulmonary infections, microcephaly, and developmental delay (Borsani et al. 2008). This patient had a de novo translocation, t(2;7), involving *TSN3* and *EXOC6B,* but it is unclear whether the *EXOC6B* variant caused the symptoms. The second patient had intellectual disability, speech delay, and facial dysmorphisms (Wen et al. 2013). This patient had a microdeletion of 2p13.2 covering *EXOC6B* and *CYP26B1*. It is not known whether EXOC6B haploinsufficiency was responsible for the symptoms. The third patient had epilepsy, intellectual disability, dysmorphisms, and autism-like behavior (Fruhmesser et al. 2013). This patient had a de novo balanced translocation t(2;8)(p13.2;q22.1) involving *EXOC6B*, and the mutation resulted in reduction in EXOC6B level. Haploinsufficiency in EXOC6B was considered the likely cause of intellectual disability in this case. The fourth patient had developmental delay, speech delay, and dysmorphisms (Evers et al. 2014). The patient had a heterozygous de novo deletion of 2p13.2 covering only *EXOC6B*. This study, together with the earlier reports, supports the notion that loss of EXOC6B function is important for intellectual disability.

### *EXOC7*

Coulter et al. described for the first time eight patients from four independent families with different pathogenic variants in *EXOC7 (Exo70)* that led to partial loss of function (Coulter et al. 2020) (Table 1). All patients had a cerebral cortex development disorder whose symptoms included developmental delay, brain atrophy, seizures, and, in severe cases, microcephaly and death in infancy. Two patients from a consanguineous family were homozygous for the c.809-2A > G splice site variant in *EXOC7*. The splice site variant disrupted *EXOC7* mRNA splicing and reduced the EXOC7 level. One patient from the second family, also consanguineous, was homozygous for the p.Ser48del variant. This region can be important for EXOC8 binding and exocyst assembly. The third family was nonconsanguineous and had two aborted fetuses with compound heterozygosity for the c. 808-2A > G splice site variant and the c.1212_1226delTGGGCTGATGCTTGA in-frame deletion variant. The fourth family was also a consanguineous family with three patients homozygous for the p.Ala523Thr variant. Since complete loss of function of EXOC7 is early embryonic lethal in mice, these patients probably had partial EXOC7 function. To further investigate the role of EXOC7 in brain development, the authors generated an *exoc7*-null zebrafish model. The embryos had small eyes, microcephaly, increased apoptosis, and early death, showing that EXOC7 is essential for development. These observations are also consistent with some of the presenting symptoms of the patients and suggest that *EXOC7* variants were responsible for this disorder.

### *EXOC8*

Whole-exome sequencing in patients with neurodevelopmental disorders of unknown cause uncovered novel candidate variants (Dixon-Salazar et al. 2012). Of 22 novel variants, a variant in *EXOC8 *(*Exo84*) was reported in a patient with Joubert syndrome, a ciliopathy disorder, for the first time (Table 1). The patient was homozygous for the c.A794T; p.E265G variant, which was located in the pleckstrin homology domain that is involved in binding phosphatidylinositol lipids for vesicle trafficking. It is unclear whether the variant is pathological for Joubert syndrome; however, given its location, the mutation might impair EXOC8 and exocyst function, which in turn might impair trafficking to cilia, resulting in ciliopathy.

In the study of *EXOC7* variants, Coulter et al. also reported three patients from a consanguineous family carrying a novel variant in *EXOC8* (2020) (Table 1). The affected children were homozygous for the p.Asp607* variant, which generates a truncated form of EXOC7. These patients also had brain atrophy, microcephaly, and developmental delay, and one patient had early death.

Another study reported six patients from a large consanguineous family with a novel variant in *EXOC8* (Ullah et al. 2022) (Table 1). The affected individuals had a neurodevelopmental disorder characterized by cerebral atrophy, microcephaly, seizures, developmental delay, and intellectual impairment. The patients were homozygous for the c.1714G > T; p.Glu572* nonsense variant, which resulted in EXOC8 truncation and was considered the pathogenic variant. The truncation is predicted to weaken its interaction with EXOC7 and, therefore, might impair exocyst assembly.

### *RALA*

De novo mutations in *RALA* were described in 10 cases in a large cohort of RASopathies (Hiatt et al. 2018) (Table 1). RASopathies, or RAS/mitogen-activated protein kinase (MAPK) syndromes, are phenotypically overlapping syndromes that include craniofacial dysmorphology, skeletal abnormalities, and neurocognitive impairment, caused by germline mutations that encode components of the RAS/MAPK signaling pathway (reviewed in Aoki et al. 2016; Kim and Baek 2019). All patients had intellectual disability, developmental delay, and facial dysmorphisms. All of the variants were in the GTP/GDP binding region of RALA: 3 c.73G > A; p.V25M, 2 c.73G > T; p.V25L, 2 c.383A > G; p.K128R, 1 c.389A > G; p.D130G, 1 c.469T > G; p.S157A, 1 c.472_474delGCT; p.A158del. All variants reduced the GTPase activity of RALA and its binding to effector proteins. Since the exocyst is an effector of RAL GTPases, it is likely to have impaired assembly, leading to impaired function of the complex in patients with *RALA* variants. Further investigation will be required to confirm this possibility. The RASopathies and neurodevelopmental disorders described in all the cases with mutations/variants in exocyst subunits have overlapping symptoms. However, RALA has other effector proteins, including phospholipase D1 (PLD1) (Yan and Thedorescu 2018). Dysregulations in PLD1 or other effectors signaling pathways might contribute to pathogenesis in patients with *RALA* variants. Therefore, the extent of the phenotype attributable to exocyst defects in patients with *RALA* variants remains unknown.

Another study analyzed a cohort of autism spectrum disorder (ASD) patients to find de novo mutations causing ASD, and a mutation in *RALA* identified in one patient was described as damaging de novo mutation in ASD (Tanaka et al. 2017; Okamoto et al. 2019) (Table 1). The patient was heterozygous for the c.73G > A; p.Val25Met variant in *RALA*, which is the same variant found in the three patients described above. The patient had severe ASD, intellectual disability, developmental delay, facial dysmorphisms, and macrocephaly. Her facial dysmorphisms are often found in Noonan syndrome RASopathy. The *RALA* variant found in this study is likely to be the cause of Noonan-like syndrome with ASD.

### Exocyst complex in neurodegeneration

Studies of exocyst subunits point to complicated involvement of the exocyst in neurobiology. *EXOC2* variants have been found in patients with developmental delay and brain abnormalities, suggesting a critical role for EXOC2 in neuronal health during development (Van Bergen et al. 2020). Surprisingly, *Sec5/EXOC2* has been identified as a genetic suppressor of poly(GR) toxicity in *Drosophila* (Lopez-Gonzalez et al. 2019). Poly(GR) is a toxic dipeptide repeat protein produced from the GGGGCC repeat expansion in the *C9ORF72* gene*. C9ORF72* mutation causes amyotrophic lateral sclerosis and frontotemporal dementia, two neurodegenerative diseases resulting in neuronal death. Furthermore, poly(GR) expression in *Drosophila* is toxic and leads to cell death, recapitulating some aspects of these diseases. Partial loss of function of Sec5/EXOC2 partially suppresses poly(GR) toxicity in *Drosophila* (Lopez-Gonzalez et al. 2019). Since EXOC2 is essential for survival and neurodevelopment, and partial loss of EXOC2 can be protective in a neurodegeneration model, EXOC2 and other exocyst subunits might have pleiotropic roles that require further investigation.

In addition, *EXOC7* mutations have been found in patients with a cortical development disorder, and in zebrafish model of loss of function of EXOC7, embryos had increased apoptosis in the telencephalon (Coulter et al. 2020). Interestingly, acting through Prpf19, EXOC7 counteracts the degradation of polyQ aggregation and mediates toxicity in spinocerebellar ataxia type 3 (SCA3) (Chen et al. 2021). SCA3 is a neurodegenerative disease caused by CAG repeat expansion in the ataxin-3 gene, resulting in polyQ aggregation. The authors showed that Prpf19 is a pre-mRNA processing factor and E3 ligase that degrades polyQ by its E3 ligase function, and thereby decreases apoptosis (Chen et al. 2021). EXOC7 was shown to bind Prpf19, and its overexpression counteracted the Prpf19 effect and increased apoptosis. Although the authors did not show whether partial loss of function of EXOC7 would reduce apoptosis or not, these two studies indicate a more complex role for exocyst subunits during neurodevelopment and neurodegeneration. Further studies are needed to explain how the exocyst might play seemingly opposite roles through temporal regulation, different binding partners, and different cellular contexts.

## Conclusion

The exocyst complex is essential for cell viability, and complete loss of function of its subunits is embryonic lethal. Mutations or variants in six exocyst complex subunits rendering the complex partially dysfunctional are pathogenic or likely to be pathogenic in neurodevelopmental disorders and ciliopathies. In most of the patients described here, common features of these disorders were intellectual disability and structural malformations in the brain. Although ciliopathies have broader clinical spectrum with multi-organ failures, renal and skeletal abnormalities appear to be prominent features of patients with exocyst mutations. Neurons and kidney cells may be especially reliant on exocyst function and hence may be more vulnerable to exocyst mutations.

Considering the role of the exocyst in exocytosis and ciliogenesis, dysfunctions in these pathways have been implicated as an underlying cause of disease. A number of studies found impairment in exocytosis and primary cilia development, and defects in protein localization to cilia in patients, providing insights into how exocyst mutations can cause pathogenesis at the molecular level. However, further studies will be necessary for deeper mechanistic understanding of exocyst dysfunction in disease.

Despite shared features at a broad level, different exocyst mutations lead to distinct disorders. Since exocyst subunits can localize to distinct subcellular localizations, mutations in different exocyst subunits might cause different phenotypes through unique pathways. Moreover, since primary cilia dysfunctions can have pleiotropic effects, the diverse phenotypes linked to exocyst mutations might be attributable to primary cilia defects. It is also worth noting that the clinical severity of ciliopathies usually correlates with cilia dysfunction and the type of underlying mutation. A mutation with strong effect such as a truncation mutation in a gene can cause MKS, which is on the severe end of the spectrum. Weaker mutations such as missense mutations in the same gene can cause Joubert syndrome, which is on the milder end of the spectrum. It would be interesting to determine whether and how the structure of the exocyst and the interactions of its subunits are altered in patients with exocyst mutations. This could help understand the impact of mutation on the function of the protein and provide further insights into genotype to phenotype correlations as well as the role of exocyst subunits in neurodevelopment and neurodegeneration.

Lastly, novel pathogenic mutations/variants in other exocyst subunits will likely be identified in neurodevelopmental disorders. Therefore, the exocyst may be a useful target of prenatal genetic screens to improve diagnostic strategies for neurodevelopmental disorders. A deeper understanding of the complex function of the exocyst in neurons and other cells can also help better understand the disease manifestations and treatment strategies.
